# The Role of B Cells in Head and Neck Cancer

**DOI:** 10.3390/cancers13215383

**Published:** 2021-10-27

**Authors:** Niki Gavrielatou, Ioannis Vathiotis, Panagiota Economopoulou, Amanda Psyrri

**Affiliations:** 1 Department of Pathology, School of Medicine, Yale University, New Haven, CT 06510, USA; niki.gavrielatou@yale.edu (N.G.); ioannis.vathiotis@yale.edu (I.V.); 2 Section of Medical Oncology, Second Department of Internal Medicine, Attikon University Hospital, National and Kapodistrian University of Athens, 12462 Athens, Greece; panagiota_oiko@hotmail.com

**Keywords:** B-cells, head and neck cancer, plasma cells, regulatory B-cells, cancer-associated antigens

## Abstract

**Simple Summary:**

Host immunity has established its role in deciding the course of cancer evolution. As cellular and molecular components in the tumor microenvironment peripherally appear to be at a constant interplay, favoring either tumor control or progression, it is vital to decrypt the immunity elements, which demonstrate the potential to be harnessed towards cancer elimination. Head and neck cancer has been characterized as densely immune infiltrated but at the same time a highly immunosuppressive malignancy due to a negative equilibrium between active and dysfunctional immune cell populations. B-cells constitute the cornerstone of humoral immunity; however, their role in cancer has been vastly overlooked in comparison to other immune subtypes and reports from multiple studies fail to show agreement on their prognostic impact. This review focuses on the role of B-cells on head and neck cancer with the aim to highlight their effect on anti-cancer immunity, as well as their possible impact on immunotherapy outcomes.

**Abstract:**

Head and neck cancer comprises a heterogenous, highly immune infiltrated malignancy, defined by a predominantly immunosuppressive tumor microenvironment (TME). In recent years, PD-1/PD-L1 immune checkpoint inhibitors have become the standard of care treatment, either as monotherapy or in combination with chemotherapy agents, thus revolutionizing the therapeutic landscape of recurrent/metastatic disease. As a result, preclinical research is increasingly focusing on TME composition and pathophysiology, aiming to comprehensively characterize the specific elements and interactions affecting anti-tumor immunity, as well as to unveil novel predictive biomarkers of immunotherapy outcomes. While T lymphocytic populations have been vastly explored regarding their effect on cancer development, B-cells constitute a far less investigated, yet possibly equally important, aspect of cancer immunity. B-cell presence, either as single cells or as part of tertiary lymphoid structures within the TME, has been associated with several anti-tumor defense mechanisms, such as antigen presentation, antibody production and participation in antibody-dependent cellular cytotoxicity, and has demonstrated prognostic significance for multiple types of malignancies. However, immunoregulatory B-cell phenotypes have also been identified both peripherally and within malignant tissue, bearing inhibitory effects on numerous immune response processes. Consequently, B-cells and their subsets demonstrate the potential to become valuable cancer biomarkers and acquire a leading role in future therapeutic strategies.

## 1. Introduction

The constantly fluctuating interactions between host immunity and cancer cells have been well established as a key component of disease control or progression, as well as a field for the development of novel anti-cancer therapeutics. The concept of immunoediting is based on these interactions and their pro or anti-neoplastic effects during its three proposed temporal phases of elimination, equilibrium and escape [1]. A large part of our existing knowledge is derived from studies focusing on the tumor microenvironment (TME), where different subsets of tumor-infiltrating or stromal immune cells and extracellular matrix molecules exhibit either pro-tumorigenic or anti-tumor qualities [2]. In certain cases, these qualities have been proven to be treatment-specific, thus rendering immunity elements into invaluable predictive biomarkers in the clinical setting and establishing the importance of tumor immune classification in addition to classical pathology assessment in directing the disease’s natural course. The mechanism of immunotherapy agents currently used for numerous malignancies depends on reinstating the activity of immune cells, predominantly T-cells, against cancer cells by blocking inhibitory pathways, with programmed cell death-1 (PD-1)/PD-L1 axis being their most prominent target. While the effect of T-cells on disease outcome has been well defined, with cytotoxic CD8+ T cell infiltration correlating with favorable outcomes in multiple studies and CD4+ subsets sustaining effective anti-tumor immunity [3,4,5], the role of B-cells is yet to be fully understood. B-cells comprise the foundation of humoral immunity, and in combination with the function of their counterpart T-cells, which rely mainly on cellular immunity, they present with critical implications in both innate and adaptive anti-cancer immune response [6,7,8]. In addition to the primary function of antibody secretion by plasma cells, which evokes antibody-dependent cellular cytotoxicity and phagocytosis, B-cells partake in a series of immune functions, including antigen-presentation and cytokine secretion within the TME, reinforcing antigen-specific immune response [9]. Reports on various malignancies, including lung, ovarian, hepatocellular, melanoma, cervical, colorectal, prostate and head and neck cancer, have demonstrated B-cell tumor infiltration as a positive prognosticator for survival [10,11,12,13,14,15,16,17]. B-cells have been reported to account for one-fourth of all infiltrating cells in some malignancies [18], and most importantly, to exhibit surface expression of PD-1, PD-L1, CTLA-4 and B-7 molecules at various levels, suggesting that their activity could also be modified by currently approved immunotherapy agents [19,20,21,22,23].

Checkpoint inhibitor-based immunotherapy targeting the programmed cell death 1 (PD-1) pathway has acquired a leading role in the management of recurrent or metastatic (R/M) Head and Neck Squamous Cell Carcinoma (HNSCC) [24]. The response rate to immunotherapy (RR) varies, as for single-agent treatment it is limited to 13–18% of patients in the total population (19% and 23% for the CPS ≥ 20, CPS ≥ 1 subgroups, respectively), and in combination with chemotherapy, RR can reach 36% in the total population (43% and 36% for the CPS ≥ 20, CPS ≥ 1 subgroups, respectively) [25,26], while autoimmune adverse events can become life-threatening without proper management. Thus, when feasible, surgical excision, followed by radiation therapy in cases of relapse, remains the foundation of treatment for this cancer [27]. In addition, a subset of immunotherapy-treated HNSCC patients has been described to paradoxically develop devastatingly rapid tumor growth following treatment initiation, an event defined as hyper-progression [28]. Given that both anticipated benefit and catastrophic immune reactions from immunotherapy are tightly linked to cellular and molecular immune composition within the TME, it becomes clear that deciphering the leverage of immune cells and their interactions on immunotherapy outcome could guide clinicians towards deciding on an individualized, optimal treatment plan for each patient. HNSCC TME presents with certain unique features in comparison with other malignancies. The distinct anatomical location of HNSCC, favoring rich lymphatic vasculature development, and the hypoxic conditions identified within these tumors, in addition to their main risk factors, including HPV positivity, smoking and alcohol consumption, lead to the formation of an “immune inflamed” yet simultaneously immunosuppressive TME [29,30,31]. In this setting, immune cell subtypes, among T-cells, tumor-associated macrophages and neutrophils, have been widely characterized for their immune-stimulatory (CD3+, CD8+, M1 macrophages, NK cells) or anti-inflammatory function (CD4+/Foxp3+ T cells, myeloid-derived suppressor cells, M2 macrophages, N2 neutrophils) with respective positive or negative effects on HNSCC outcomes [32,33,34], while B-cells have only recently attracted researchers’ interest with inconclusive results regarding their prognostic role, so far [35]. 

The present review focuses on the effect of B-cells and their subpopulations in head and neck cancer in respect to their association with disease outcome and the future perspectives for their incorporation in therapeutic decisions. 

## 2. B Cells in Anti-Tumor Immunity

### 2.1. Prognostic Value of Tertiary Lymphoid Structures

B-cells are primarily found in tumors either as cell aggregates in the invasive margin or as components of tertiary lymphoid structures (TLS), and in some cases, as scarce intratumoral cells. Malignant tissues are the source for the production of both tumor-specific (TSA) and tumor-associated antigens (TAA), with the former being selectively expressed on cancer cells as the result of newly acquired somatic mutations, as opposed to the latter, which can also be found in normal tissue [36]. T-cell priming and B-cell proliferation and clonal expansion occur within secondary lymphoid organs (SLO), following the uptake and presentation of tumor antigens by dendritic cells. Resembling the structural and functional characteristics of SLO, tertiary lymphoid structures (TLS) constitute well-organized ectopic lymphocytic aggregates formed under chronic inflammatory conditions within the TME, which perpetually induce local immune responses [37]. B-cells are found in germinal-center-like formations in TLS and participate in the reactivity against neighboring cancer cells after differentiation into memory B-cells and antibody-producing plasma cells. The prognostic significance, as well as the association of TLS presence with clinicopathologic characteristics, varies immensely among different cancer types [38]. Results from studies in gastric cancer have depicted the positive impact of TLS on survival [39,40], while TLS have also been linked to advanced disease in the same tumor type [41]. TLS were deemed a favorable prognosticator in a study on triple-negative breast cancer [42], while in another report, they were associated with higher tumor grades [43]. In ovarian cancer, TLS presence, as was defined by colocalization of CD20+ B-cells with CD8+ cytotoxic T-cells, has been associated with improved survival outcome, an effect that was not replicated by CD8+ T-cell presence alone [44]. Additionally, a 12-chemokine signature (CCL2, 3, 4, 5, 8, 18, 19, 21, CXCL9, 10, 11, 13) associated with TLS formation has been correlated with improved prognosis in melanoma, colorectal and breast cancer [45,46,47]. Similarly, multiple reports have demonstrated the positive prognostic value of TLS in NSCLC, where these formations were hypothesized to sustain durable anti-tumor immune response and even facilitate adaptive immunity activation independently of SLOs [48,49,50,51]. Finally, TLS have also been suggested to reduce the risk of early recurrence in hepatocellular carcinoma [52]. TLS have also been found to have positive prognostic significance in HNSCC, as was reported by Li and colleagues. In this study, multiplex immunofluorescence and immunohistochemistry assays were employed for the characterization of TLS in HNSCC TME, and 26.8% of the cohort population exhibited TLS presence. TLS positive cases showed association with prolonged overall and recurrence-free survival (RFS) as opposed to TLS negative cases (*p* = 0.005, HR:3.784; 95% CI 1.498–9.562 and *p* = 0.014, HR:3.296; 95% CI 1.279–8.490 for OS and RFS, respectively), independently of other known prognosticators, while TLS presence in combination with CD8+ T-cell and CD57+ NK cell density was found to have the highest predictive accuracy. TCGA data analysis confirmed that higher expression of TLS-related gene signatures was also associated with improved OS, and remarkably, TLS were identified at a high percentage in peritumoral dysplastic tissue areas suggesting a possible implication in the early stages of HNSCC carcinogenesis [53]. Increased BCL-6+/CD21+ intratumoral germinal center formations have also been described in stage I NSCLC in comparison with higher disease stages supporting the above findings in HNSCC [54]. Given that TLS have been established as an essential component of anti-tumor immunity, significant research efforts have been made towards deciphering their potential ability to predict response to immunomodulatory cancer therapies. Petitprez et al. indicated that highly B-cell-infiltrated TLS in the TME of soft-tissue sarcomas correlated with improved PFS and enhanced response rate to PD-1 blockade treatment [55]. Moreover, transcriptomic analysis performed by Helmink et al. showed that B-cells, as well as TLS, in the TME of metastatic renal cell carcinoma and advanced stage melanoma are associated with response to immune checkpoint blockade (ICB) treatment [56]. Finally, Cabrita et al. discovered a distinct TLS-associated gene signature predictive of survival outcomes in an ICB treated melanoma cohort. In the same study, increased B-cell presence in TLS correlated with high levels of TCF7+ naïve and memory T-cells in contrast to dysfunctional T-cell phenotypes, which dominated the TME in the absence of TLS [57]. The above findings underscore the need to investigate TLS as potential predictive biomarkers of response to immunotherapy in other malignancies as well, including HNSCC.

### 2.2. Prognostic Role of B-Cells Outside TLS

In a meta-analysis on the prognostic role of tumor-infiltrating CD20+ B-cells (TIL-B) and plasma cells across 19 different malignancies, including HNSCC, TIL-Bs and plasma cells, demonstrated an overall positive prognostic impact, in agreement with CD3+ and CD8+ cell density and contributed to the improved prognostic effect of increased T-cell presence. The analysis also showed either a positive or neutral prognostic effect of TIL-Bs in ovarian, breast, gastric, hepatocellular, soft tissue sarcoma, esophageal and biliary tract cancer while in NSCLC, colorectal, melanoma pancreatic and HNSCC, evidence of TIL-B prognostic significance were contradicting [58]. Griss et al. characterized plasmablast-like cells in the TME of human melanoma tissue as an inflammation-sustaining subgroup, vital for the recruitment of CD8+ cytotoxic T-cells, as well as the enhancement of response to anti-PD-1 blockade agents [59]. Genomic characterization of immune cell elements among multiple malignancies revealed a positive correlation of a 60-gene B-cell signature with OS in NSCLC. The same study illustrated increased BCR diversity, independently of gene segment expression, as a positive survival prognosticator in melanoma, while the opposite effect was observed in renal cell carcinoma [60]. De Falco et al. also identified increased levels of plasmablasts in peripheral blood samples of metastatic, non-progressive melanoma, NSCLC and renal cells carcinoma patients. Further investigation of this patient subgroup revealed the presence of persistent B-cell clones undergoing progressive class switching, suggesting a selective anti-tumor antibody response against specific neo-epitopes [61]. The research focused on the prognostic effect of B-cells in HNSCC has also delivered inconclusive results, suggesting intra-patient, as well intra-tumoral, immune heterogeneity. HNSCC demonstrates increased B-cell infiltration, and specific phenotypes have been identified within TME. Flow cytometry analysis of B-cell surface expression markers, performed on HNSCC tumor tissue, isolated PBMCs and healthy oral mucosa samples, revealed significantly higher levels of CD86+ activated and CD86+/CD21- antigen-presenting B-cell phenotypes in tumor samples compared with PBMCs and non-cancerous mucosa, while memory B-cells characterized by IgD−/CD27+ phenotype were increased in HNSCC patients’ tumor tissue and peripheral blood as opposed to healthy donors. In the same study, by Lechner et al., CD27+/CD38hi/CD20− plasmablast were also higher in tumor samples and HNSCC PBMCs than in healthy mucosa, while CD27+/CD38hi/CD138hi/CD20− plasma cells showed a differential localization in tumor tissue rather than PBMCs [17]. Pretscher and colleagues demonstrated the association of increased peritumoral CD20+ B-cell presence in HNSCC metastatic lymph nodes with prolonged disease-free survival [62], a finding that was confirmed by an additional study by Suárez-Sánchez et al., where CD20+ primary-tumor-infiltrating B-cells were associated with improved disease-specific survival [63]. The positive prognostic impact of CD20+ B-cells has also been indicated in an analysis of TCGA quantitative proteomics and transcriptomics data where high levels of expression of *MS4A1*, the gene encoding for CD20, were correlated with increased overall survival as opposed to CD20 protein expression, which failed to demonstrate statistical significance [64]. CD20+ B-cell density has also been associated with lower T-stage in HNSCC, suggesting a potential role of B-cells taming cancer progression in earlier disease stages [65], although other studies found no correlation of B-cell density with stage, indicating the need for investigation of this hypothesis in larger patient cohorts [17]. The importance of B-cells in preventing cancer development has been illustrated in a long-term follow-up study on the effects of B-cell depletion after anti-CD20 (Rituximab) treatment, where a lack of B-cells was linked to the development of secondary malignancies [66]. Nonetheless, different reports have described a pro-tumorigenic effect of B-cells on HNSCC. B-cell depletion by anti-CD20 mAb treatment resulted in augmented responsiveness to chemotherapy in murine squamous carcinomas, which was attributed to increased CD8+ recruitment under the influence of macrophage-secreted CCR5 [67]. In contrast, the response to PD-1 blockade has been reported to be unaffected by B-cell depletion [68]. De Visser et al. proposed a mechanism by which B-cells might participate in tumorigenesis in premalignant, chronic inflammatory tissue samples from HPV16+ mice. The authors concluded that activated B-cells can acquire the role of the distal orchestrator of innate immunity by immunoglobulin production, which influences tissue-resident immune cell functions and results in the formation of immune complexes [69]. A subsequent study by Andreu and colleagues reported similar findings, supporting the hypothesis of B-cells’ implication in de novo carcinogenesis [70]. Accordingly, in castration-resistant prostate cancer, tumor-infiltrating B-cells have been suggested to drive tumor progression via lymphotoxin production [71].

### 2.3. Direct Cytotoxicity and Antibody-Dependent Cell-Mediated Cytotoxicity

Antigen presentation has been described as yet another important physiologic function of B-cells, as they exhibit the ability to drive T-cell expansion and memory formation after initial priming by dendritic cells, as well as to participate in antigen cross-presentation to other APCs [72,73]. Bruno et al. demonstrated that activated tumor-infiltrating B-cells in NSCLC demonstrate the capacity to present antigens to CD4+ T-cells and transform them into a highly activated phenotype [74]. Furthermore, B-cells have been found to engage in direct cytotoxicity and are essential for antibody-dependent cell-mediated cytotoxicity (ADCC). Hagn et al. described the CD40 ligation-dependent B-cell differentiation into granzyme B-producing cells under the influence of IL-21, suggesting that B-cells undertake a cytotoxic role in the absence of adequate antigen-specific T-cell activation, as occurs in early tumorigenesis [75]. An additional mechanism of B-cell direct cytotoxicity has been proposed by Tao et al.; the authors reported that B-cells expressing FasL prompted the death of Fas+ tumor cells employing Fas/FasL pathway in a murine breast cancer model [76]. ADCC is based on the interaction of antibodies—primarily of the IgG, IgA and IgE classes—coating target cells, with Fc receptors found on the surface of effector cells—mainly NK cells, but also monocytes, neutrophils, eosinophils and dendritic cells. This interaction, which results in the phagocytosis-independent death of target cells, has become the cornerstone for the development of anti-cancer targeted therapies using artificially synthesized monoclonal antibody agents [77] and at the same time constitutes an important physiologic mechanism of B-cell anti-tumor activity. Gilbert et al. identified the production of tumor-antigen-specific IgG antibodies by mature B-cells isolated from peripheral blood of melanoma patients. B-cell cultures from melanoma patients had the ability to produce antibodies targeting cancer cells, in contrast to healthy controls, leading to disease control by ADCC [78].

## 3. HPV-Specific B-Cell Implications

Infection with a high-risk HPV variant, mainly HPV 16 and 18, is etiologically linked with 38,000 newly diagnosed HNSCC cases worldwide, with the highest prevalence observed in developed countries, most prominently in North America and Europe [79]. HPV-related HNSCC is regarded as a distinct disease, demonstrating favorable prognosis most commonly attributed to its characteristic molecular oncogenic patterns and immune landscape [80]. Importantly, HPV infection has been described to drive the production of specific TAAs, which stimulate a robust anti-tumor response, as well as TME architecture and composition. Regarding B-cell infiltration, a differentially expressed gene signature characterizing B-cells has been identified for HPV-related HNSCC, while T-cell immune signature showed no difference in respect to infection status [81]. Russel and colleagues identified increased CD20+ B-cell presence in HPV+ as opposed to HPV- cases, although increased CD20+ presence was not significantly correlated with survival in that cohort [82]. Nonetheless, results from two independent research groups indicate that the improved prognosis of HPV-related disease might be linked to higher B-cell infiltration in addition to their cross-reaction with T-cells. In the first study, Hladíková et al. reported higher B-cell density among HPV+ tumors and described a positive prognostic association of increased CD20+ B-cell density, as well as of CD20+/CD8+ cell-to-cell interaction in the HPV-related subgroup [83]. Additional findings from the comparative transcriptomic analysis of immune cell composition between HPV positive and negative tumors showed that high infiltration with memory B-cells, which are chemotactically attracted to the TME following CXCL13 production by CD4+ T-cells, correlated with improved outcomes in HPV-related tumors [84]. Recently, Wieland and colleagues reported that antibody-producing cells located in the TME of HPV-related tumors give rise to HPV antigen-specific antibodies, with E2 viral protein being their most common target. Moreover, they characterized an activated HPV-antigen-specific memory B-cell phenotype within the TME, consistent with chronic HPV infection and identified the presence of multiple antibody-secreting, activated and germinal center B-cells organized in clusters mainly in tumor-stroma [85]. Kim et al. reported the association of B-cell gene expression with the prolonged OS using RNA-sequencing analysis on HPV+ HNSCC. In the same study, exposure of an HPV+ murine model to PD-1 inhibition and radiotherapy led to increased proliferation of B-cells, plasma cells and antigen-specific B-cells, as well as to the expansion of B-cell germinal center formation [86]. Exploring the potentially distinct effect of B-cell phenotypic variation and localization within the TME among HPV-related and HPV-negative tumors, Ruffin et al. revealed that the former are characterized by naïve and germinal center B-cells, while the latter are predominantly infiltrated by memory B-cell subpopulations and plasma cells. Furthermore, within the HPV-positive group, the presence of TLS enriched with germinal center B-cells correlated with improved OS and importantly, extensive infiltration with this specific B-cell phenotype was associated with longer PFS irrespective of TLS formation [87]. In concordance with the theory that distinct phenotypic subpopulations, rather than overall B-cell presence in TLS facilitate anti-tumor response, a recent study on cutaneous melanoma metastasis concluded that AID+ B-cells undergoing somatic hypermutation correlated with improved OS, as opposed to mature CD21+ B-cells, which conferred worse survival outcome [88].

## 4. Immunity Impeding B-Cell Phenotypes

### 4.1. Regulatory B-Cells

Regulatory B-cells (Bregs), a heterogenous population of B-cells characterized by the expression of a variety of surface markers, have also been investigated regarding their activity and cell-to-cell interactions within the TME [89]. Bregs were first identified and investigated in autoimmune disease [90], chronic inflammatory and allergic conditions [91,92,93] and solid organ transplantation as IL-10-producing cells with various phenotypes, which promote Treg development and mitigate effector CD4+ and CD8+ T-cell activity [94]. While Bregs primarily drive immunosuppression, discordant results from multiple studies indicate that their effect on cancer evolution demonstrates a dual nature, tipping the scale either towards disease control or progression depending on their various phenotypes (Table 1). Over a decade ago, Bregs were identified as the potential mediators of squamous carcinogenesis under the influence of TNF-a in murine models [95]. Additionally, a distinct IL-10-producing B-cell sub-phenotype named “B1” has been shown to drive macrophage polarization towards the immunosuppressive M2 phenotype in vitro [96], while CD1dhiCD5+ Bregs have been implicated in the downregulation of T-cell-mediated inflammation in mice [93]. Moreover, in healthy individuals, CD19+CD24hiCD38hi Bregs have been shown to restrain autoimmunity by inhibiting TH1 and TH17 T-cell differentiation and promoting the development of Tregs [97]. Another subset of tumor-infiltrating Bregs characterized by CD19+CD38+CD1d+IgM+CD147+ phenotype has been reported to demonstrate granzyme B expression after IL-21 activation and subsequently contribute to T-cell suppression via TCR degradation and overexpression of regulatory molecules (IL-10, CD25, IDO) [98]. Furthermore, B7-H1^High^ CD81^High^ CD86^High^ CD62L^Low^ IgM^Int^ Bregs expressing Stat3 have been implicated in enabling metastatic tumor growth following TGFβ-dependent differentiation of CD4+ T-cells into Tregs [99]. Importantly, results from a study on a 4T1 murine breast cancer model showed that CD20+ B-cell depletion by anti-CD20 mAb infusion provoked preferential enrichment with CD20LowCD137Low regulatory B-cells leading to tumor progression and metastasis, thus demonstrating the essential role of B-cells in disease control [100].

**Figure 1:**
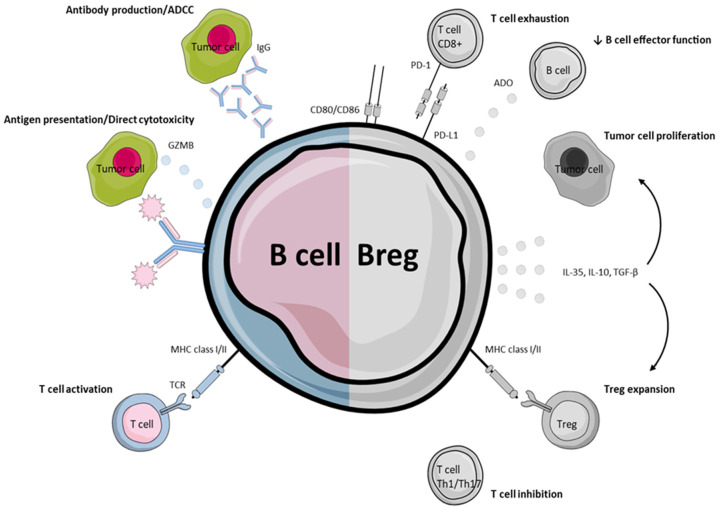
Demonstration of major B-cell functions within the TME. Effector B-cells, plasmablast and plasma cells enhance anti-tumor immunity by tumor-specific antibody production, ADCC, antigen presentation and T-cell activation (Left). Bregs, on the other hand, promote immunosuppression by T-cell exhaustion, effector B-cell inhibition, Treg expansion and tumor proliferation via inhibitory cytokine production. ADCC: antibody-dependent cell-mediated cytotoxicity; GZMB: granzyme B; TCR: T-cell receptor; MHC: major histocompatibility complex; ADO: adenosine; Th1: T helper cell type 1/17; TGF-β: Transforming growth factor-beta.

Furthermore, findings on the function and protein expression of certain Breg phenotypes have illustrated them as eligible targets for ICB therapy. In hepatocellular carcinoma, a distinct phenotype of CD5(hi)CD24(−/+)CD27(hi/+)CD38(dim) Bregs displaying high levels of PD-1 expression has been observed to exert pro-tumorigenic effects and T-cell suppression through IL-10 production after activation of the PD-1/L1 pathway [101]. Moreover, in a study on systemic lupus erythematosus CD19+CD24hiCD38hi Bregs have been shown to impede Th1 differentiation following IL-10 production, an effect that was overcome by the addition of CD80 and CD86 mAbs, which are known ligands for the CTLA-4 immunoregulatory molecule [107]. The same phenotype was investigated by Wang et al. in gastric carcinoma, where it was found to promote immunosuppression by the reduction of IFN-γ and TNF-α secretion by CD4+Th cells and was associated with CD4+FoxP3+ Treg density [108]. An additional study on EMT-6 mammary tumor implanted in mice revealed that B-cells acquire a regulatory phenotype (LAP/TGF-β1, CD80, CD86, PD-L1) after cell-to-cell interaction with cancer cells and their immunosuppressive dynamic can be counteracted by mAbs against TGF-β and PD-L1, leading to tumor shrinkage [102]. In metastatic melanoma, PD-L1+-circulating B-cells were associated with the advanced disease stage and have increased presence in metastatic rather than primary sites. This subpopulation presented a naïve-like phenotype (CD20+CD27-), high IgM and IgD production compared with total B-cells and most importantly, suppressed T-cell activity through PD-L1 expression [103]. A similar Breg phenotype (PD-1-PD-L1+CD19+) has been described to be acquired by B-cells under the influence of MDSCs in breast cancer TME [109]. Bregs have also been investigated as potential therapeutic targets for various other agents in preclinical studies, with the aim of overcoming their immunoinhibitory effects and reinstating efficient anti-tumor immunity. The use of resveratrol in a 4T1 metastatic breast cancer murine model achieved shrinkage of lung metastasis, a result that was attributed to Breg inhibition, and consequently, TGF-β downregulation, through inactivation of Stat3 [110]. Similarly, in the same tumor type, inactivation of 5-lipoxygenase/leukotriene/PPARa pathway by MK886 Breg inhibitor resulted in a significant reduction of tumor growth and elimination of its metastatic potential [111]. The most important effector B-cell and Breg functions in the TME are illustrated in Figure 1.

### 4.2. Regulatory B-Cells in HNSCC

In HNSCC, Breg function has also been shown to result in the abrogation of T-cell anti-tumor activity; however, results on their prognostic effect remain contradicting. CD24hi/CD38hi/CD19+ Bregs were found in higher density in comparison with CD19+ B-cells in HNSCC TME and this regulatory phenotype preferentially localized in tumor tissue rather than in PBMCs isolated from the same cases [17]. Jeske and colleagues recently reported the effects of an adenosine (ADO)-producing Breg subpopulation characterized by CD39 and CD73 surface marker expression using human HNSCC tissue samples and murine squamous carcinoma models. Cells of this specific phenotype were found to be preferentially located within the TME in comparison with peripheral blood and promoted immunosuppression by inhibition of B effector cells’ function, mediated by the downregulation of Bruton’s tyrosine kinase phosphorylation by ADO [104]. Additionally, Zhou et al. described a CD19+IL-10+ Breg subpopulation in tongue squamous cell carcinoma, which promoted the differentiation of CD4+ T-cells into Tregs and correlated with reduced OS, an effect that was dependent on Treg density in multivariate analysis [105]. Opposite results were reported in a study by Nourouzian et al., where atypical memory B-cells (CD27–IgM–IgD–) and B-regs presenting a CD24hiCD38hi phenotype isolated from non-sentinel lymph nodes (LNs) of HNSCC patients were associated with an absence of LN cancer infiltration and lower histological grade, both known as good disease prognosticators [106]. Although contradicting, the above results can be interpreted as the reflection of the prognostic variability of Bregs depending on specific phenotypes and tissue localization. 

## 5. Conclusions

HNSCC tumor biology and TME composition have been investigated in depth in an effort to recognize the precise elements that decide the fate of tumor development, and at the same time, to identify specific components susceptible to manipulation in favor of successful treatment. With immunotherapy gaining the leading role in advanced HNSCC treatment, tumor-infiltrating immune cells have been put under the spotlight in search of the optimal balance for cancer obliteration. Current research evidence suggests that B-cells constitute a pivotal player in anti-tumor immunity and exhibit the potential for modulation towards disease control and/or elimination in multiple types of malignancies, including HNSCC. Notably, as distinct B-cell phenotypes evoke either immunity-stimulating or pro-tumorigenic effects, either directly or via interaction with other immune cells, it is essential to acquire a comprehensive understanding of their mechanisms of action and specify targets among B-cell-related markers that could contribute to treatment response, depending on their activation or inhibition by therapeutic agents.

## Figures and Tables

**Table 1:** Breg phenotypes in cancer.

Breg Phenotype	Location	Tumor Type	Function/Effect
CD19+CD38+CD1d+IgM+CD147+	Tumor tissue	Multiple solid tumors	T-cell inhibition/exhaustion [98]
Stat3, B7-H1High CD81High CD86High CD62LLowIgMInt	Tumor tissue	4T1 breast cancer murine model	CD4+ Treg expansion/metastatic tumor growth [99]
CD20LowCD137Low	Tumor tissue	4T1 breast cancer murine model	Tumor progression/metastasis [100]
CD5(hi)CD24 (−/+)CD27(hi/+)CD38(dim)	Tumor tissue	HCC	T-cell exhaustion through PD-1/PD-L1 pathway [101]
LAP/TGF-β1, CD80, CD86, PD-L1	Tumor Tissue	EMT-6 breast cancer murine model	Immunosuppression, tumor progression [102]
CD20+CD27−, IgMhi and IgDhi	PBMCs	Melanoma	T-cell suppression through PD-1/PD-L1, association with advanced stage and metastasis [103]
CD39+CD73+, ADOhi	Tumor tissue, PBMCs	HNSCC	Effector B-cell suppression [104]
CD19+IL-10+	Tumor tissue	HNSCC	Promotion of CD4+ Treg differentiation [105]
CD24hiCD38hi	Tumor draining LNs	HNSCC	Absence of LN tumor infiltration/Low grade [106]

PBMC: peripheral blood mononuclear cells; HCC: hepatocellular carcinoma; GC: gastric cancer, LN: lymph node.

## Data Availability

The data presented in this study are available in the present article.
